# Emergence of New Delhi Metallo-*β*-Lactamase (NDM) Genes Detected from Clinical Strains of *Escherichia coli* Isolated in Ouagadougou, Burkina Faso

**DOI:** 10.1155/2023/4813225

**Published:** 2023-06-02

**Authors:** Boukaré Kaboré, Henri S. Ouédraogo, Oumarou Zongo, Ganamé Abasse Ouédraogo, François Tapsoba, Sanogo Bougma, Koudbi Jacob Zongo, Boukaré Zeba, Yves Traoré, Idrissa Sanou, Aly Savadogo

**Affiliations:** ^1^Department of Biochemistry and Microbiology, Laboratory of Applied Biochemistry and Immunology, University Joseph KI-ZERBO, 03 BP 7021, Ouagadougou 03, Burkina Faso; ^2^Department of Biochemistry and Microbiology, Faculty of Applied Science and Technology, University of Dedougou, BP 176, Dedougou, Burkina Faso; ^3^UFR Health Sciences, University Joseph KI-ZERBO, 03 BP 7021, Ouagadougou 03, Burkina Faso; ^4^Laboratory of Bacteriology and Virology at Tengadogo University Hospital, 11 BP 104 Ouaga CMS 11, Ouagadougou, Burkina Faso

## Abstract

The emergence and spread of carbapenem resistance in Gram-negative bacilli such as *Klebsiella pneumoniae*, *Escherichia coli*, *Acinetobacter baumannii*, and *Pseudomonas aeruginosa* through the production of carbapenemases is a global phenomenon. It threatens patient care and leads to therapeutic impasses. This study aims to genotypically determine the prevalence of the most frequent carbapenemase genes among multidrug-resistant *E. coli* strains isolated from patients at a biomedical analysis laboratory. A total of fifty-three unduplicated *E. coli* strains isolated from patient samples with a multidrug-resistant (MDR) profile were subjected to polymerase chain reaction (PCR) testing for carbapenem resistance genes. This study allowed us to identify fifteen strains carrying resistance genes among the fifty-three *E. coli* strains. All fifteen strains produced the metallo-*β*-lactamase enzymes; this represents a rate of 28.30% of study strains. Among these strains, ten carried the NDM resistance gene, NDM and VIM genes were detected in three strains and VIM was identified in two strains of *E. coli*. However, carbapenemases A (KPC and IMI), D (OXA-48), and IMP were not detected in the strains studied. Thus, NDM and VIM are the main carbapenemases detected in the strains in our study.

## 1. Introduction

*Escherichia coli* (*E. coli*) is a versatile microorganism; it is a well-known commensal of the normal gut microbiome that can sometimes also be a very virulent and often deadly pathogen [1]. *E. coli* is the most frequent cause of urinary tract infections (UTI) in Burkina and is also involved in other infections such as bloodstream infections and infections of surgical wounds [2–4]. The uncontrolled use of antibiotics in recent years to fight human bacterial infection has led to selection pressure in pathogen and commensal bacteria [5]. Thus, in *E. coli,*multidrug-resistant (MDR) strains have emerged. Indeed, many antibiotic resistance genes have been described in *E. coli* such as beta-lactam resistance genes TEM, SHV, CTX-M, and quinolone resistance genes (qnr) [6–8]. The most clinically important resistance is that linked to carbapenems, which are beta-lactams of last resort used for the treatment of serious bacterial infections [9]. Several carbapenem resistance genes have been described in *E. coli* [10]; NDM-1 (New Delhi metallo-beta-lactamase-1) is the most recently discovered transferable molecular class B. It was first described in *Klebsiella pneumoniae* and *E. coli* isolated in Sweden in 2008 from a patient transferred from a New Delhi hospital [11].

*Enterobacteriaceae* are responsible for a large proportion of nosocomial infections and are associated with significant morbidity and mortality; the emergence and dissemination of resistance to carbapenems pose a major public health problem. So, information of carbapenemase-producing bacteria study is limited in Burkina Faso. Knowledge of the types of resistance genes will guide the choice of appropriate antibiotic therapy. It will make it possible to formulate recommendations on the use of antibiotics as well as the implementation of infection control strategies. This study aimed to determine genotypically the prevalence of genes encoding resistance to carbapenems in *E. coli* species. This involved looking for carbapenemases: *Klebsiella pneumoniae* carbapenemase (KPC), Imipenem-hydrolyzing-*β*-lactamase (IMI), active on imipenem (IMP), New Delhi metallo-*β*-lactamase (NDM), Verona integron-encoded for metallo-*β*-lactamase (VIM), and oxacillinase (OXA-48) in *E. coli* multidrug-resistant isolated in Ouagadougou, Burkina Faso.

To carry out the study, strains presenting a multidrug-resistance profile were collected and carbapenem sensitivity test was conducted on Mueller–Hinton agar (MHA) using disc method. Then, bacterial DNA extracts were made for the PCR reaction on strains showing resistance or reduced sensitivity to carbapenems.

## 2. Materials and Methods

### 2.1. Bacterial Isolates

**Figure 1:**
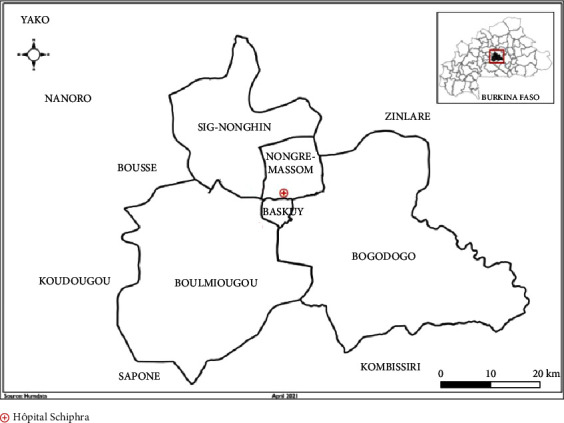
Health map of the city of Ouagadougou showing the sampling site at the biomedical laboratory of the Schiphra hospital. The city is divided into five health districts, and our sample collection site is in the Nongre-Massom health district.

This was a retrospective study of clinical strains collected after routine analysis. A total of 53 unduplicated strains of *E. coli* multidrug-resistant (MDR) were collected from cultured urine and pus specimens at Schiphra Hospital Medical Analysis Laboratory, from inpatient and outpatient specimens (Figure 1), from February to September 2020. Most of the strains collected came from urine culture and only four strains were isolated from pus (*E. coli* P80, *E. coli* P66, *E. coli* P46, *E. coli* P49) culture. Clinical strains were identified by standard bacteriological methods using Gram stain and biochemical method using API 20E kits (bioMérieux, Marcy-l'Étoile, France) gallery. Pure strains were inoculated into Luria Bertani broth supplemented with 20% glycerol and stored at −20°C for subsequent tests and analyses.

### 2.2. Antimicrobial Susceptibility Testing

Antibiotic sensitivity was performed on Mueller–Hinton agar medium (MH) according to the CA-SFM/EUCAST guidelines [12]. A bacterial suspension corresponding to 0.5 McFarland was inoculated by swab on MH medium, then the antibiotic discs were deposited. After 18–24 hours of incubation at 37°C, the reading was carried out and the strains were classified susceptible, intermediate, or resistant with respect to the critical diameter according to CA-SFM/EUCAST guidelines. Following antibiotics were tested on the strains in routine examination: Fosfomycin, Norfloxacin, Gentamicin, Cotrimoxazole, Ciprofloxacin, Levofloxacin, Ceftriaxone, Cefixime, Chloramphenicol, Cefuroxime, Imipenem, Amikacin, Ticarcillin-Clavulanate, Ceftazidime, Meropenem, Cefepime, Ticarcillin, Aztreonam, Netilmicin, Nitrofuran, and Amoxicillin-Clavulanate. The susceptibility profile to the different antibiotics of all the strains is presented by Kaboré et al. [4]. These antibiotics were used in our study because they are the first-line antibiotics used for bacterial infections. However, carbapenems were used in the study to determine resistance to these so-called last-resort antibiotics for Gram-negative infections. After the collection of the bacterial strains, the antibiogram was performed on the strains with carbapenems, imipenem, meropenem, and ertapenem (company Liofilchem, Italy). When resistance to carbapenems has been found, the Imipenem-EDTA and Imipenem-boric acid test were performed for observation of the inhibition diameter, this for phenotypic detection of metallo-beta-lactamase and carbapenemase class A [13].

### 2.3. Molecular Characterization for Detection of Carbapenemase-Encoding Genes

#### 2.3.1. DNA Extraction

A total of 2-3 identical colonies of *E. coli* were resuspended in 250 *μ*L of distilled sterile water in 1.5 mL Eppendorf tube. This was boiled at 100°C for 10 min and immediately frozen at −20°C for 10 min, centrifuged at 13000 rpm for 5 min, and the supernatant containing DNA was stored at −20°C for further using [14–17]. The presence of DNA was assessed by qualitative analysis in agarose gel electrophoresis.

#### 2.3.2. Molecular Identification of Carbapenemase Genes

All strains of *E. coli* were tested for PCR using oligonucleotide primers specific for the carbapenem resistance genes listed in Table 1. Genes were amplified by polymerase chain reaction (PCR) on a Mastercycler nexus gradient (Eppendorf, flexlid). Reaction mixture consisted of a total volume of 25 *μ*L; composed of 4 *μ*L of Master Mix (Inqaba Biotec), 2 *μ*L of primers (Forward and Reverse), 17 *μ*L of nuclease-free water, and 2 *μ*L of DNA extract. PCR reagents were provided by Inqaba Biotec West Africa, Nigeria.

Amplification reactions were performed in a thermal cycler (Mastercycler nexus gradient) according to the following program: initial denaturation at 95°C for 5 minutes, denaturation at 95°C for 45 seconds, annealing at specific primer temperature (Table 1), extension at 72°C for 1 minute, and final extension at 72°C for 10 minutes for 35 cycles. *Pseudomonas aeruginosa ATCC 27853* was used in the PCR cycle and used as a negative control for the carbapenemase genes sought.

After PCR amplification, 4 *μ*L of each reaction were separated by 1.2% agarose gel electrophoresis for 120 min at 80 V in TAE 1X buffer in the migration tank (ENDURO™ GEL XL). DNA was stained with ethidium bromide (1 *μ*g/mL) and amplified DNA bands were visualized using a UV transilluminator (UVP PhotoDoc-lt Imaging System).

## 3. Results and Discussion

### 3.1. Antibiotic Testing

All the strains were tested on several classes of antibiotics including beta-lactams and proved to be multiresistant (MDR) [4]. The phenotypic profile of carbapenemase-producing strains detected by polymerase chain reaction is shown in Table 2.

**Figure 2:**
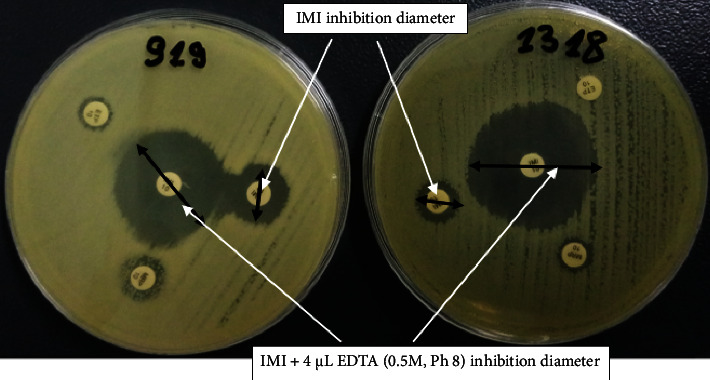
Phenotypic detection of metallo-beta-lactamases by combined disk test for *E. coli* 919 and *E. coli* 1318.

Figure 2 shows the phenotypic profile of metallo-beta-lactamase production observed when these strains are carbapenemases-producing type MBL.

Inhibition zone ≥7 mm with the Imipenem-EDTA disk was compared to the Imipenem disk alone and was considered as MBL positive [24, 25].

It is difficult to demonstrate resistance to carbapenems by the disc diffusion method. In our study, only two (*E. coli* 1318 and *E. coli* 919) strains showed resistance by disc diffusion method, while the PCR technique revealed 15 strains showing the presence of carbapenemase genes. In fact, according to the CA-SFM [12], any strain with reduced sensitivity to at least one of the carbapenems must therefore be considered suspicious of carbapenem production. Furthermore, the CA-SFM recalls that Ertapenem is the carbapenem with the best sensitivity for the detection of carbapenem-producing strains. Thus, any strain showing reduced sensitivity to Ertapenem (MIC >0.5 mg/L or an inhibition diameter (10 *μ*g/ml disk) <25 mm) by the agar diffusion test can be subjected to the screening algorithm for carbapenemase-producing strains. Given the results of sensitivity to carbapenems, it should not be assumed that the strains (*E. coli* 1473, *E. coli* 1476, and *E. coli* 1321) can be detected as carbapenemase producers because these strains present a phenotypic sensitivity to carbapenems and to Ertapenem considered carbapenem of choice for the detection of carbapenemase-producing strains. The average age of the patients is 60.21 years with extremes of 11 years and 85 years. Male sex is predominant at 64.28% (*n* = 9) with extreme ages 60 years and over except one male patient who is 42 years old. At these ages, infections are more frequent with recurrent antibiotic treatments resulting in selection pressure for resistant strains.

### 3.2. Carbapenemase Genes

**Figure 3:**
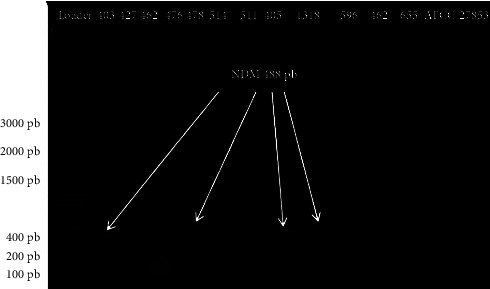
Agarose gel electrophoresis of NDM gene amplicons (488 bp).

**Figure 4:**
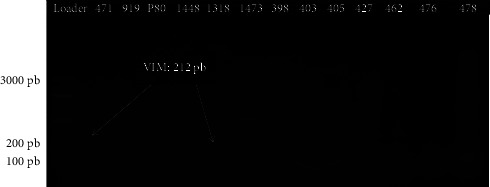
Agarose gel electrophoresis of VIM gene amplicons (212 bp).

Among the strains of *E. coli*, including 49 from urinary infections and 4 from pus, we found ten strains whose PCR reaction products showed bands corresponding to the bands of the NDM genes at 488 bp, which represents a rate of 18.87% and PCR products from two strains showed bands at 212 bp corresponding to VIM. However, we found that the PCR products of three strains showed two bands at both 488 bp and 212 bp (NDM and VIM); these are *E. coli* 1318, *E. coli 514,* and *E. coli 583* strains (Table 3). Indeed, the PCR did not reveal the presence of any bands for the genes corresponding to base pairs 481 (IMI), 796 (KPC), 744 (OXA-48 likes), and 432 (IMP). No bands corresponding to a carbapenemase gene were observed with the PCR products of the DNA extracts of the strains from pus samples, namely: *E. coli* P80, *E. coli* P66, *E*. *coli* P46, and *E. coli* P49.  Figure 3 shows the band profile of the NDM resistance genes visualized with the UVP PhotoDoc-lt imaging system after migration, and Figure 4 shows the band profile of the VIM resistance genes.

Table 3 shows the result of the genotypic investigation of the resistance genes sought, with (−) indicating that the gene sought was not detected and (+) indicating that the gene sought was detected.

The presence of carbapenemase-producing bacteria in hospitals poses a serious challenge. The problem with carbapenemases is that most of the resistance genes are located on mobile elements and are therefore easily transferred from one bacterial species to another [26–28]. Indeed, out of 53 multidrug-resistant* E. coli* strains, we identified 15 strains producing metallo-beta-lactamase genes, of which ten harboured the NDM gene, two strains harboured the VIM gene, and three harboured both genes at the same time (NDM and VIM). All these strains came mainly from urinary infections (49 strains were of urinary origin and 4 came from pus). The rate of carbapenemase-producing* E. coli* among the multidrug-resistant strains was 28.30%, which confirms the multidrug-resistant nature of these strains. In our study, only carbapenemase B (NDM and VIM) were detected in *E. coli* strains, which could explain their rapid diffusion within the bacterial strains. Other resistance genes were not researched and the strains could harbour these genes, hence their multidrug-resistance; since these genes have already been described in *E. coli* in Burkina Faso (TEM, SHV, CTX-M,…), so genes for resistance to other families of antibiotics (quinolones, aminoglycosides, etc.) were not sought. However, previous studies had revealed a high rate of extended-spectrum beta-lactamases among clinical strains in Burkina Faso [6, 7, 29]. In West Africa, the prevalence of ESBL varies from one country to another; thus, rates of 49.4% and 63.4% to 96% have been reported respectively in Ghana and Mali in hospitals and in the community [30]. Our study revealed the presence of DNA bands at 488 bp for NDM and 212 bp for VIM of the PCR reaction products of *E. coli* strains. Indeed, the presence of carbapenemase genes of the NDM, VIM, and OXA type had been detected in strains of *E. coli* and other Gram-negative bacilli [29]. An OXA-181-like carbapenemase was detected in four species of *E. coli* for the first time in Burkina Faso [31]. These authors have been able to demonstrate the plasmids on which these genes are located, namely IncX3 for the OXA-181 type genes and *In*cF for the NDM-1 genes. In West Africa, few studies have been reported on the resistance of carbapenemase-producing Gram-negative bacilli. However, some case studies in Togo, Mali, and Nigeria have reported the presence of OXA and NDM genes [32–34]. NDM, OXA, and VIM genes have been reported in most African countries [35]. The movement of populations for economic and health reasons could explain the rapid spread of the NDM gene around the world; indeed, travelers to endemic areas are at risk of exposure to resistant pathogens and are likely to return colonized [36, 37]. Described in *Klebsiella pneumoniae* isolated from a Swedish patient of Indian origin with a urinary tract infection in New Delhi, India, and designated NDM-1 [12], more than twenty NDM variants have been described to date [38]. The genetic variability of the NDM gene could explain its worldwide distribution. Asian continent serves as the major reservoir of NDM producers, with around 58.15% abundance of NDM-1 variant distributed mostly in China and India [39].

#### 3.2.1. Limit of the Study

We did not have the opportunity to confirm the expression of carbapenemase genes by DNA sequencing technique.

## 4. Conclusion

Our study highlighted the presence of resistance genes NDM and VIM among the clinical strains of *E. coli* in Burkina. NDM and VIM enzymes hinder the action of beta-lactams and other antibiotics, resulting in therapeutic impasses. Therefore, increased surveillance should prevent the spread of these resistance genes, which are a major concern worldwide. Finally, a structural study of these enzymes could make it possible to develop new molecules in association with beta-lactams to combat these resistances.

## Figures and Tables

**Table 1:** Primers used for detection of carbapenemases genes in *E. coli*.

Target	Sequence (5′ ⟶ 3′)	Annealing temp. °C	Amplicon size (bp)	References
IMI	F: CTACGCTTTAGACACTGGC	57	481	[16]
	R: AGGTTTCCTTTTCACGCTCA			

KPC	F: CTGTCTTGTCTCTCATGGCC	60	796	[18–20]
	R: CCTCGCTGTRCTTGTCATCC			
OXA-48	F: TTGGTGGCATCGATTATCGG R: GAGCACTTCTTTTGTGATGGC	58	744	[18]

IMP	F: GTTTATGTTCATACWTCG	45	432	[21, 22]
	R: GGTTTAAYAAAACAACCAC			

NDM	F: TGGCAGCACACTTCCTATC	58	488	[23]
	R: AGATTGCCGAGCGACTTG			

VIM	F: AGTGGTGAGTATCCGACAG	52	212	[23]
	R: TCAATCTCCGCGAGAAG			

**Table 2:** Phenotypic outcome for carbapenem antibiotics from carbapenemase-producing strains.

Bacterial strains	Inhibition diameter (mm)			Patient's age (years)/sex
	Imipenem (10 *μ*g)	Meropenem (10 *μ*g)	Ertapenem (10 *μ*g)	
*E. coli 471*	24	24	23	70/M
*E. coli 919*	15	00	00	62/M
*E. coli 1318*	10	00	00	11/F
*E. coli 1473*	28	30	30	79/M
*E. coli 1476*	27	30	29	29/F
*E. coli 403*	25	28	25	79/M
*E. coli 405*	26	27	26	85/M
*E. coli 513*	27	28	26	Not specified
*E. coli 514*	29	24	15	42/M
*E. coli 583*	29	29	27	77/M
*E. coli 596*	24	29	27	25/F
*E. coli 1321*	28	28	30	72/F
*E. coli 1831*	25	26	21	60/M
*E. coli 469*	26	26	24	69/F
*E. coli 543*	26	26	25	83/M
CA-SFM acceptable limit for *E. coli* ATCC 25922	26–32	28–35	29–36	
Target for *E. coli* ATCC 25922	29	31-32	32-33	

**Table 3:** Antibiotic resistance genes screened in *E. coli* strains.

Species	Carbapenemase A		Carbapenemase B		Carbapenemase D	
	bla_KPC_	bla_IMI_	bla_NDM_	bla_IMP_	bla_VIM_	bla_OXA-48_
*E. coli 471*	−	−	−	−	**+**	−
*E. coli 919*	−	−	**+**	−	−	−
*E. coli 1318*	−	−	+	−	**+**	−
*E. coli 1473*	−	−	**+**	−	−	−
*E. coli 1476*	−	−	**+**	−	−	−
*E. coli 403*	−	−	**+**	−	−	−
*E. coli 405*	−	−	**+**	−	−	−
*E. coli 513*	−	−	-	−	**+**	−
*E. coli 514*	−	−	**+**	−	**+**	−
*E. coli 583*	−	−	**+**	−	**+**	−
*E. coli 596*	−	−	**+**	−	−	−
*E. coli 1321*	−	−	**+**	−	−	−
*E. coli 1831*	−	−	**+**	−	−	−
*E. coli 469*	−	−	**+**	−	−	−
*E. coli 543*	−	−	**+**	−	−	−

(+): resistance gene detected; (−): resistance gene not detected.

## Data Availability

The data used to support the findings of this study are available from the corresponding authors upon request.
